# Molecular Identification and Functional Characterization of the Fatty Acid- and Retinoid-Binding Protein Gene *Rs-far-1* in the Burrowing Nematode *Radopholus similis* (Tylenchida: Pratylenchidae)

**DOI:** 10.1371/journal.pone.0118414

**Published:** 2015-03-03

**Authors:** Chao Zhang, Hui Xie, Xi Cheng, Dong-Wei Wang, Yu Li, Chun-Ling Xu, Xin Huang

**Affiliations:** 1 Laboratory of Plant Nematology and Research Center of Nematodes of Plant Quarantine, Guangdong Province Key Laboratory of Microbial Signals and Disease Control / Department of Plant Pathology, College of Natural Resources and Environment, South China Agricultural University, Guangzhou, People’s Republic of China; 2 Institute of Genetic Engineering, Southern Medical University, Guangzhou, Guangdong, People’s Republic of China; Russian Academy of Sciences, Institute for Biological Instrumentation, RUSSIAN FEDERATION

## Abstract

Fatty acid- and retinoid-binding protein (FAR) is a nematode-specific protein expressed in the nematode hypodermis. It is involved in nematode development, reproduction, and infection and can disrupt the plant defense reaction. In this study, we obtained the full-length sequence of the *far* gene from *Radopholus similis* (*Rs-far-1*), which is 828 bp long and includes a 558 bp ORF encoding 186 amino acids. A protein homology analysis revealed that *Rs*-FAR-1 is 75% similar to *Mj*-FAR-1 from *Meloidogyne javanica*. A neighbor-joining phylogenetic tree was inferred and showed that *Rs*-FAR-1 is most similar to *Pv*-FAR-1 from *Pratylenchus vulnus*. A fluorescence-based ligand-binding analysis confirmed that *Rs*-FAR-1 can combine with fatty acids and retinol. qPCR was used to assess *Rs-far-1* expression levels at different developmental stages in different *R*. *similis* populations, and its expression was 2.5 times greater in the highly pathogenic *Rs*-C population than in the less pathogenic *Rs*-P population. The highest expression was found in females, followed by eggs, juveniles and males. When *R*. *similis* was treated with *Rs-far-1* dsRNA for 36 h, the reproduction and pathogenicity decreased significantly. *In situ* hybridization revealed *Rs-far-1* transcripts in the *R*. *similis* hypodermis. Additionally, *R*. *similis* treated with *Rs-far-1* dsRNA or water were inoculated into *Arabidopsis thaliana*. Allene oxide synthase (AOS) expression in *A*. *thaliana* was upregulated during early infection in both treatments and then returned to the expression levels of the control plant. Compared with the control plant, AOS expression significantly decreased in *A*. *thaliana* inoculated with water-treated *R*. *similis* but significantly increased in *A*. *thaliana* inoculated with *Rs-far-1* dsRNA-treated *R*. *similis*. This finding indicates that *Rs-far-1* regulates AOS expression in *A*. *thaliana*. *Rs*-FAR-1 plays a critical role in *R*. *similis* development, reproduction, and infection and can disturb the plant defense reaction. Therefore, *Rs-far-1* is an important target gene to control *R*. *similis*.

## Introduction

The burrowing nematode *Radopholus similis* [(Cobb, 1893) Thorne, 1949] is a migratory endoparasite that was first discovered in banana root in Fiji in 1891. *R*. *similis* is known to attack over 250 plant species and is distributed throughout most tropical and subtropical areas, where it severely harms bananas, citrus crops, peppers and many other economically important crops [1,2]. It also has been known to invade ornamental plants from the genus *Anthurium*, and it causes severe economic losses in Holland and other countries that export large volumes of flowers [3–5]. The economic damage inflicted by *R*. *similis* has caused this species to be listed as a quarantine plant pest by many countries and as one of the top 10 most economically important nematode species[6–8].

Methods for effectively controlling *R*. *similis* are continuously under development. Currently, the research focus has transferred to molecular biology approaches [3,9–11], rather than traditional approaches [12–16]. The esophageal gland secretion proteins of plant-parasitic nematodes (PPNs) are widely considered to be closely related to their pathogenicity [17], and pathogenic genes have been the focus of many studies [3,8,18–21]. In addition to esophageal gland secretion proteins, the body wall of PPNs is involved in the complicated interaction between nematodes and their hosts. Integumentary secretion proteins produced by PPNs play an important role in breaking through the plant defense system and invading host plants. Cloning the genes of integumentary secretion proteins and determining their functions are important for elucidating the host invasion processes of plant nematodes and will likely provide a new approach to control PPNs [22]. Fatty acid- and retinoid-binding protein (FAR), which is an integumentary secretion protein located in the cuticle of PPNs, may play a key role in the host invasion process of plant nematodes. Moreover, PPNs require fatty acids and retinoids to synthesize lipids, in addition to macromolecular compounds related to the development of eggs and to maintaining physiological activity [23]. Iberkleid et al. [24] reported that overexpression of *Mj-far-1* in the hairy roots of tomatoes caused significant downregulation of the proteinase inhibitor *Pin2* (accession no. L21194) and γ-thionin (accession no. AJ133601.1) and inhibited the defense response. However, nematodes are not able to produce fatty acids and retinoids themselves; instead, they depend on FARs to bind lipids from the environment or a host plant to survive[25,26].

FARs may also inhibit the plant defense reaction by impeding the expression of genes related to the jasmonic acid (JA) pathway in the host plant. JA induces the plant defense reaction as well as the synthesis of linolenic and linoleic acid, which are important precursors in the JA pathway. PPNs decrease the precursor concentration and the expression of other related genes in the JA pathway by binding to linolenic and linoleic acid in the host plant and inhibiting the plant defense reaction. In addition, linolenic acid participates in redox reactions catalyzed by allene oxide synthase (AOS) to produce jasmonates[24,27]. Therefore, the expression of *far*, in addition to the inhibition of linolenic acid, may be associated with the expression of the AOS gene.

In this study, based on ESTs obtained from a previous investigation involving a suppression subtractive hybridization (SSH) library between different pathogenic populations of *R*. *similis*[9], the full-length sequence of *Rs*-*far-1* was amplified using RACE. The RNAi technique was employed to test the relationship between *Rs*-*far-1* expression and the pathogenicity and fertility of *R*. *similis*; qRT-PCR was used to test *Rs*-*far-1* expression in *R*. *similis* treated with *Rs*-*far-1* dsRNA as well as *Rs*-*far-1* expression during the different developmental stages of *R*. *similis*; *in situ* hybridization was performed to localize *Rs*-FAR-1; and tissue culture techniques employing *A*. *thaliana* cultures and RNAi were applied to test the relationship between *Rs*-*far-1* from *R*. *similis* and the AOS gene from *A*. *thaliana*.

## Materials and Methods

### Plant materials

The tested cultivars of *Anthurium andraeanum* Alabama were purchased from the Flowers and Plants Research Center in Guangzhou, Guangdong. The roots of the anthurium seedlings were washed with sterile water, and nematodes were collected on nested 0.147 mm- and 0.026 mm-pore sieves. Baermann funnels were used to separate the nematodes from the precipitate, and a microscopic inspection was performed [28]. If nematodes were present in the roots, the roots were soaked in hot water at 62°C to remove the nematodes [15]. All of the uncontaminated seedlings were grown in sterilized soil medium for 15 days for later use. The flowerpots employed in this study were 14 cm in diameter, with a volume of 1.5 L, and 1.2 L of sterilized soil was added to each pot.

### Nematodes and extraction methods

Two populations of *R*. *similis*, designated *Rs*-C and *Rs*-P, were collected from the roots of the ornamental plants *Calathea makoyana* and *Philodendron cv* Green Emerald, respectively. The reproduction and pathogenicity of the *Rs*-C population are significantly higher than those of the *Rs*-P population[9]. The length of the ITS sequences of both *Rs-*C and *Rs-*P was 625 bp (Accession numbers: JQ619539, JQ619538). There were two variant nucleotides in the ITS sequence at 5.8 s that differed between *Rs-*C and *Rs-*P, at NO.373 and NO.402, respectively. The populations were cultured on excised carrot (*Daucus carota*) disks in Petri dishes with a 6 cm diameter in a 25°C incubator [29,30]. Nematode extraction was performed according to Zhang et al. (2012) [9].

#### RNA extraction, cDNA synthesis and generation of the complete *Rs-far-1* sequence

Total RNA extraction and microelute total RNA extraction were performed according to Zhang et al. (2012) [9]. The candidate *Rs-far-1* fragment was screened out of the library through an alignment analysis. To obtain the complete *Rs-far-1* sequence, 3’ RACE primers (NEST-S1 and NEST-S2) (Table 1) and 5’ RACE primers (NEST-A1 and NEST-A2) (Table 1) were designed using UPM and NUP (SMART RACE cDNA amplification kit) to amplify the 3’ and 5’ ends of *Rs-far-1*, respectively, employing the SMART RACE cDNA amplification kit (Clontech, Takara Biotechnology (Dalian) Co., Ltd., Dalian, China). Finally, we spliced three *Rs-far-1* fragments (5’ end, middle fragment and 3’ end) into the complete *Rs-far-1* sequence. Two specific primers (cds-F and cds-R) (Table 1) were designed based on the spliced complete *Rs-far-1* sequences to amplify the complete *Rs-far-1* sequence.

**Table 1:** Primers used in this research.

Primer name	Sequence	Source
**Actin-F**	5′-GAAAGAGGGCCGGAAGAG-3′	Jacob et al. (2007)
**Actin-R**	5′-AGATCGTCCGCGACATAAAG-3′	Jacob et al. (2007)
**NEST S1**	5′-CCGAACCTGGAGGAACTGCG-3′	
**NEST S2**	5′-CGCAACCTGGTCAAGGGCAAGA-3′	
**NEST A1**	5′-GAATGCCTTCGCGTCCGGGTTC-3′	
**NEST A2**	5′-CTCGTCGGTGAGTTCGTTGTA-3′	
**qPCR-F**	5′-AACTCACCGACGAGGACAAGGCCG-3′	
**qPCR-R**	5′-GATCTTGCCCTTGACCAGGTTGC-3′	
**cds-F**	5′-CCTATCCATCGCATTTGAGACTC-3′	
**cds-R**	5′-CTTCTGCTTAGGCGGCGGTGG-3′	
**FAR-T7S**	5′-TAATACGACTCACTATAGGGACTCACCGACGAGGACAAGGC CG-3′	
**FAR-A**	5′-GATCTTGCCCTTGACCAGGTTGC-3′	
**FAR-T7A**	5′-TAATACGACTCACTATAGGGGATCTTGCCCTTGACCAGGTTG C-3′	
**FAR-S**	5′-AACTCACCGACGAGGACAAGGCCG-3′	
**GFP-T7S**	5′-GGATCCTAATACGACTCACTATAGGGCACAAGTTCAGCGTGTCCGGCG-3′	Zhang et al. (2012)
**GFP-A**	5′-CGATGCGGTTCACCAGGGTGTCG-3′	Zhang et al. (2012)
**GFP-T7A**	5′-GGATCCTAATACGACTCACTATAGGGCGATGCGGTTCACCAGGGTGTCG-3′	Zhang et al. (2012)
**GFP-S**	5′-CACAAGTTC AGCGTGTCCGGCG-3′	Zhang et al. (2012)
**IN-T7A**	5′-TAATACGACTCACTATAGGGCCTCAAGAGCCTGGTCCTCGGTC–3′	
**IN-S**	5′-AACTCACCGACGAGGACAAGGCCG-3′	
**IN-T7S**	5′-AATACGACTCACTATAGGGAACTCACCGACGAGGACAAGGCCG-3′	
**IN-A**	5′-CCTCAAGAGCCTGGTCCTCGGTC—3′	
**AOS-S**	5′- CGGTTACGGCTCAATACGG-3′	
**AOS-A**	5′- ATCTCTCCGGCACAAACTCAT-3′	
**At-ACTIN-F**	5′- CGTTGCTGTTGGTGTTATTAAGAG-3′	Bae et al. (2009)Bae et al. (2009)
**At-ACTIN-R**	5′- GAGGGAGAGAGAAAGTCACAGAAAT-3′	

### *Rs-far-1* sequencing and alignment analysis

DNAssist 2.2 and Clustal X were used for the sequencing and alignment analyses. The sequences were aligned against a non-redundant protein database (nr) and a non-redundant nucleotide database (nt) using the BLASTN and BLASTX programs available via NCBI (http://www.ncbi.nlm.nih.gov/). The amino acid alignment analysis was performed using Swiss-Prot and TrEMBL (http://us.expasy.org/). RPS-BLAST was employed for performing a conserved domain analysis in NCBI; the Signal P 3.0 Server (http://www.cbs.dtu.dk/services/SignalP/) was used to predict protein signal peptides; and the transmembrane structure was predicted with TMPred (http://www.ch.embnet.org/cgi-bin/TMPRED/). A phylogenetic tree of FARs from different PPNs was constructed using the neighbor-joining method [18] with MEGA4.0 (Molecular Evolutionary Genetics Analysis, USA) and BioEdit V7.0.0.

### Expression and purification of recombinant *Rs-*FAR-1 and ligand binding experiment

To obtain the purified *Rs*-FAR-1 protein, full-length *Rs-far-1* was amplified from the plasmid and cloned into the prokaryotic expression vector pET-32a (Novagen, Madison, WI, USA). The plasmid was transformed into *Escherich*. *coli* DH5α for sequence confirmation. The recombinant plasmid DNA was then introduced into *E*. *coli* BL21 (DE3) for expression. The expression of the recombinant protein was examined via sodium dodecyl sulfate-polyacrylamide gel electrophoresis (SDS-PAGE) with Coomassie brilliant blue staining after treatment with 1 mM isopropyl β-D-thiogalactopyranoside (IPTG). The fluorescent analogs 11-(5-dimethylaminonaphthalene-1-sulfonyl amino) undecanoic acid (DAUDA) (Sigma, USA), cis-parinaric acid (Molecular Probes) (Cayman, USA), retinoic acid (Sigma, USA) and oleic acid (Sigma, USA) were used to measure the binding activity of the purified *Rs-*FAR-1. Fluorescence was measured as previously described [26], and the excitation wavelengths employed for DAUDA, retinoic acid and cis-parinaric acid were 345, 350 and 319 nm, respectively. The dissociation constants (Kd) for the binding of *Rs-*FAR-1 to DAUDA and cis-parinaric acid were estimated by adding increasing concentrations of *Rs-*FAR-1 to DAUDA or cis-parinaric acid (10 mM in PBS). Increasing concentrations of the fluorescent ligands were added to a 10 mM *Rs*-FAR-1 solution in Tris-HCl buffer to measure the Kd of *Rs*-FAR-1-retinoid binding activity. The results of the fluorescence analyses were adjusted to a single noncompetitive binding model using standard nonlinear regression techniques (Microcal ORIGIN software). The estimates of Kd and Fmax are indicated.

### Expression of *Rs-far-1* and RNAi efficiency

qPCR was used to assess the variation in the expression levels of *Rs-far-1* between the *Rs-*C and *Rs-*P nematode populations and among different developmental stages in *Rs-*C, including females, males, juveniles and eggs. *Rs-*C and *Rs-*P were isolated from carrot calli and subjected to total RNA extraction. RNA samples were prepared from 100 nematodes at each developmental stage using the MicroElute total RNA kit (OMEGA) according to the manufacturer’s instructions. A total of 100 females, males, juveniles and eggs were used for RNA extraction. The quantity and quality of the obtained RNA were then checked using a Nano-drop spectrophotometer, after which the RNA was stored at −80°C for further analysis. All of the RNA samples used for qPCR were prepared three times as three biological replicates.

Total RNA was then treated with RQ1 RNase-Free DNase (Promega, Beijing, China) for 15 min at 37°C, as described by Zhang et al. [9]. cDNA was synthesized using the ReverTra Ace qPCR RT kit (TOYOBO Life Science, Shanghai, China) according to the manufacturer’s instructions. Based on the complete sequence of *Rs-far-1*, the specific primers qPCR-F and qPCR-R (Table 1) were designed to determine *Rs-far-1* expression. According to the method described by Jacob et al. [10], the primers Actin-F and Actin-R were synthesized (Table 1) to amplify the reference gene β-actin. qPCR was performed on mixed life stages of *Rs-*C and *Rs-*P and on the females, males, juveniles, and eggs of *Rs-*C. The reactions were performed in triplicate in a CFX-96 (Bio-Rad) qPCR machine using the SYBR Green qPCR Master Mix-plus kit (TOYOBO) according to the manufacturer’s protocol, with the following reaction conditions: 95°C for 15 s and 60°C for 30 s (40 cycles). The initial data analysis was carried out using Bio-Rad CFX-96 manager software, which generated Ct values and extrapolated the relative levels of PCR products from standard curves. Melt curves were obtained routinely, which allowed the possibility of both contamination and primer dimers to be discounted. Actin was used as a positive control in all experiments. All experiments were performed in triplicate.

### Localization of *Rs-far-1* using *In situ* hybridization

*In situ* hybridization was performed as previously described [26,31]. In total, 10,000 mixed-stage nematodes, including females, males and juveniles, were isolated from carrot calli and fixed in 3% paraformaldehyde at 4°C for 16 h. Specific sense (IN-T7S, IN-A) and antisense (IN-T7A, IN-S) (Table 1) primers were designed to amplify DIG-labeled sense and antisense RNA probes (Roche, Germany) from the full-length *Rs-far-1* cDNA. The DIG-labeled sense and antisense RNA probes were added to hybridization solution containing nematode sections and then rotated at 47°C for 12 h. Following hybridization, the nematodes were examined, and photographs were obtained using differential interference microscopy.

### Synthesis of *Rs-far-1* dsRNA

An approximately 200 bp *Rs-far-1* dsRNA fragment was amplified using specific primers (FAR-T7S, FAR-A, FAR-T7A and FAR-S) (Table 1) with a T7 promoter [9]. The quantity of dsRNA was measured with a NanoDrop spectrophotometer, and the dsRNA was analyzed through 1.2% agarose gel electrophoresis. Finally, the dsRNA was diluted to 2.0 μg/μl and stored at -80°C for later use. Non-endogenous control dsRNA (125 bp, green fluorescent protein gene, *gfp*) was generated using specific primers (G-T7S, G-A, G-T7A, and G-S) (Table 1).

In total, 5,000 nematodes from the *Rs*-C population were collected and transferred to an Eppendorf tube, treated with DEPC water, and soaked in 50 μl of the *Rs-far-1* dsRNA solution (2 μg/μl) at room temperature for 12 h, 24 h or 36 h. A solution containing non-endogenous *gfp* dsRNA (2 μg/μl) was used as a control. The treatment times applied for the control were same as those used for the *Rs-far-1* dsRNAs. Additionally, untreated nematodes were employed as a blank control. The treated nematodes were cleaned three times with DEPC water, and total RNA was then extracted. qPCR was performed to analyze the suppression of the *Rs-far-1* transcript in the nematodes following RNAi treatment. Each experiment was performed using three biological replicates. After RNAi treatment, RNA was extracted, and qRT-PCR was performed to analyze *Rs-far-1* expression.

### Reproduction and pathogenicity of nematodes after RNAi treatment

In total, 30 female *Rs*-C nematodes were treated with the *Rs-far-1* dsRNA solution (2 μg/μl) for 12 h, 24 h or 36 h. The controls were treated with the *gfp* dsRNA solution (2 μg/μl) for 12 h, 24 h or 36 h or without any dsRNA solution. Another control group of 30 female *Rs-*P nematodes that were not treated with a dsRNA solution was also included. All of the nematodes were then inoculated onto a carrot callus and maintained at 25°C in a dark incubator for 56 days, after which the nematodes in the carrot callus were isolated, and the total number of nematodes was calculated as previously described [9]. Each treatment was repeated five times.

The pathogenicity of the *Rs-far-1* RNAi-treated nematodes, the *gfp* dsRNA solution-treated nematodes, and the untreated nematodes was compared. Each treatment was adjusted to a suspension containing 100 nematodes per ml. Three small holes (5 cm depth) were made in the soil with a 0.5 cm diameter glass rod for the anthurium stem, and 10 ml of the nematode suspension was pipetted into the soil. The plants were managed according to the usual practices, but they were not watered for the first 5 days. Five replicates were performed for each treatment. After 56 days, the nematodes were extracted from the roots and soil of all of the treated plants and then counted and calculated as described above. In addition, the above-ground plant weight, root weight, and disease severity were recorded. Disease severity was classified according to Zhang et al. [9].

### Influence of *Rs-far-1* on AOS expression in *A*. *thaliana*

#### In vitro culture of *A*. *thaliana*

Surface sterilization of the *A*. *thaliana* seeds and *R*. *similis* was performed according to Sindhu et al. [21]. The *A*. *thaliana* seeds were sterilized through the following steps: washing with 10% sodium hypochlorite and 0.01% Tween-20 for 3 min; washing with 70% ethanol for 1 min; and washing with ddH_2_O 3 times. Then, 7–10 *A*. *thaliana* seeds were inoculated at 9 cm in sterilized MS media. The media were then cultured in an incubator at a constant temperature (28°C for 16 h and 20°C for 8 h), and 7 days later, germinative *A*. *thaliana* seeds were prepared for subsequent use.

#### Preparation of *R*. *similis* and inoculated *A*. *thaliana*

In total, 20,000 nematodes from the *Rs-*C population were collected from the cultured excised carrot disks: 10,000 nematodes were directly surface sterilized, and the other 10,000 nematodes were first treated with *Rs-far-1* dsRNA and then surface sterilized. The sterilized nematodes were mixed with 100 μl of 1.5% low melting agarose. This method produces a more uniform nematode distribution and enables more effective inoculation. The nematode solution was added to the rhizosphere of the *A*. *thaliana* roots, at a density of 300 nematodes per dish. There were 3 treatments: inoculated untreated nematodes; inoculated RNAi-treated nematodes; and untreated *A*. *thaliana*, which was used as a blank control. Each treatment was repeated 5 times (5–7 *A*. *thaliana* in each plate).

#### qRT-PCR analysis of AOS expression after inoculation

An inverted microscope (NIKON ECLIPSE Ti-V) was used to observe the inoculation results 12 h after inoculation. Three *A*. *thaliana* roots were cut off and placed in three 1.5 ml Eppendorf tubes at 12 h, 24 h, 36 h, 48 h, 60 h and 72 h after inoculation. The roots were washed using DEPC water and stored at -80°C for later use. Untreated *A*. *thaliana* roots were used as a blank control. Each treatment was repeated 3 times.

Microeluted RNA was extracted from each treated *A*. *thaliana* root using the MicroElute total RNA kit (OMEGA) according to the manufacturer’s instructions. cDNA was synthesized with the ReverTra Ace qRT-PCR RT Kit (TOYOBO) according to the manufacturer’s instructions. Specific qRT-PCR primers were designed according to Bae et al. (2009) [32], and qRT-PCR was performed to assess AOS expression in each of the treatments.

### Data analysis

All of the data obtained in this study were subjected to analysis of variance (ANOVA), and multiple comparisons of the means were performed using Duncan’s Multiple Range Test at *p =* 0.05 using SAS (Release 8.01).

## Results

### Cloning and sequence analysis of full-length *Rs-far-1*

Based on the ESTs obtained from a previously reported *R*. *similis* SSH library [9], specific *Rs-far-1* primers were designed, and the UPM/NUP adaptor from the SMARTer RACE cDNA Amplification kit (Clontech) was used to amplify the 5’ and 3’ ends of *Rs-far-1*. Fragments with lengths of 350 bp and 556 bp were amplified, and an approximately 800 bp sequence was obtained after alignment and splicing. Based on the 800 bp sequence, specific CDS primers were designed to amplify the full-length *far-1* sequence, and an 828 bp sequence containing the 5’ UTR, ORF and 3’ polyA was obtained. According to the NCBI ORF finder, the 828 bp sequence includes a 558 bp open reading frame (ORF) encoding 186 amino acids; the 5’ UTR contains 16 nucleotides; and there are 142 nucleotides between the 3’ terminator and the polyA sequence. The amino acids sequence of the conserved FAR domain was predicted and aligned. A conserved domain spanning from amino acids 29–179 of FAR-1 was identified. The results indicate that FAR-1 belongs to the fatty acid- and retinoid-binding (FAR) family of proteins, and the FAR-1 gene was designated *Rs-far*-*1*. The sequences of *Rs*-FAR-1 and FARs from other nematodes were downloaded from NCBI and used to conduct BLAST and homology analyses. *Rs*-FAR-1 and *Mj-*FAR-1 from *M*. *javanica* presented the highest homology (75%), and it was predicted that these two FAR-1s share a similar function. *Rs*-FAR-1 also showed high similarity with *Gp*-FAR-1 from *Globodera pallida* (74%) and showed 59%, 56%, 55%, 53%, 52%, 50%, 42% and 43% similarity with the FAR-1s of *Aphelenchoides besseyi*, *Onchocerca ochengi*, *Litomosoides sign*, *Brugia pahangi*, *Wuchereria bancrofti*, *Ascaris suum*, *Caenorhabditis elegans*, and *C*. *briggsae*, respectively.

**Fig 1:**
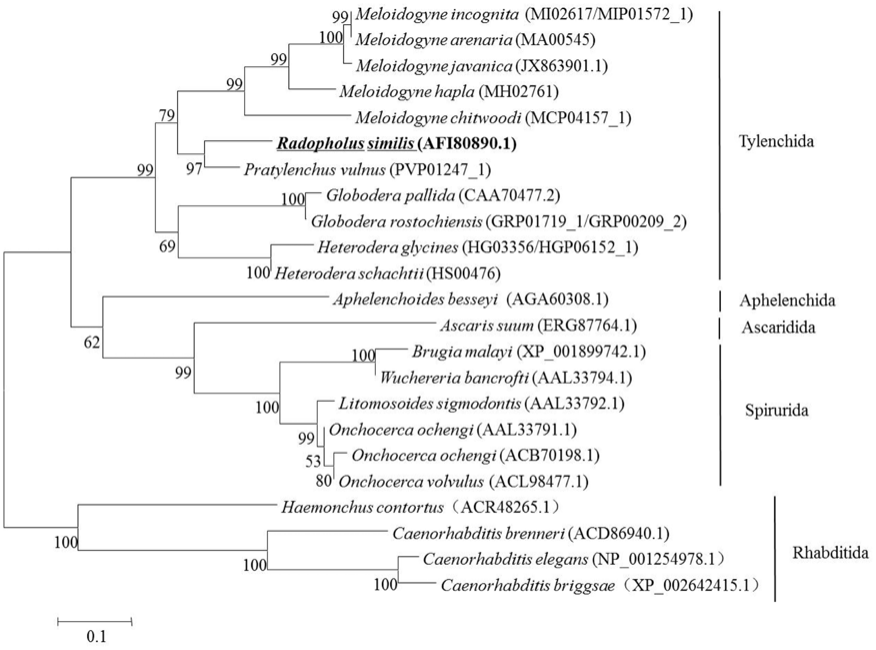
Neighbor-joining phylogenetic tree of 23 fatty-acid and retinol-binding proteins (FAR). The tree for 23 fatty-acid and retinol-binding proteins (FAR) from 22 species of nematodes was generated using MEGA version 4 (Tamura *et al*, 2007)

A phylogenetic tree was constructed using the *Rs*-FAR-1 amino acid sequence and the sequences from 23 other FAR-1 proteins from 22 nematode species (Fig. 1). It was found that *Rs*-FAR-1 from *R*. *similis* and *Pv*-FAR-1 from *Pratylenchus vulnus* clustered together, belonging to the same Tylenchida order, suggesting that they exhibit a close genetic relationship. In addition, all of the 23 FAR proteins from the 22 nematode species were divided into 5 groups: Tylenchida, Aphelenchida, Ascaridida, Spirurida and Rhabditida.

### Ligand binding

**Fig 2:**
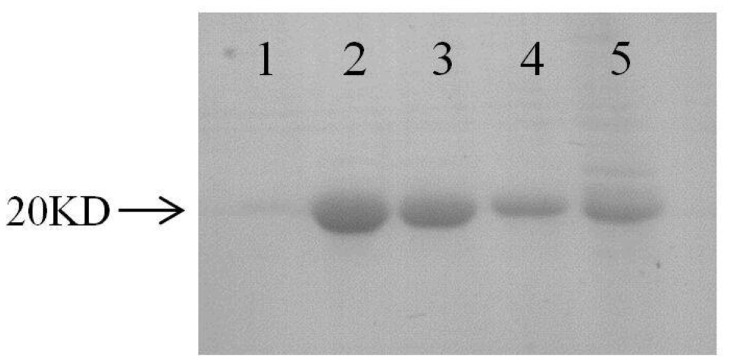
SDS-PAGE of the *Rs-*FAR-1 recombinant protein. Lane 1 shows the negative control; Lanes 2–5 correspond to *Rs-*FAR-1 recombinant protein.

**Fig 3:**
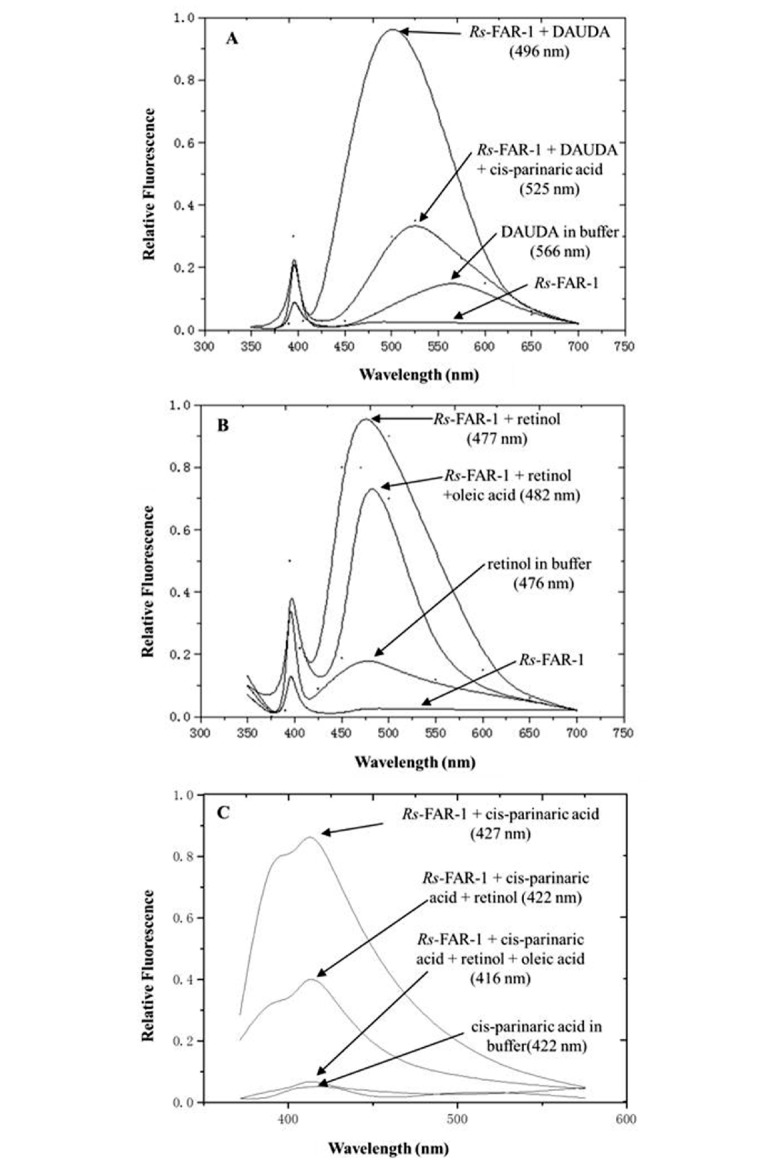
Ligand binding by r*Rs*-FAR-1. (A) Fluorescence emission spectra (excitation at 345 nm) of DAUDA either alone or after the addition of r*Rs*-FAR-1. A reverse change in DAUDA emission was observed after the addition of oleic acid to the r*Rs*-FAR-1+DAUDA complex. The wavelengths of peak emission by DAUDA were different. (B) Fluorescence emission spectra (excitation at 350 nm) of retinol in ethanol either alone or after the addition of r*Rs*-FAR-1. A competitive effect of oleic acid was also observed. (C) Fluorescence emission spectra (excitation at 319 nm) of cis-parinaric acid either alone or after the addition of r*Rs*-FAR-1. The effects of the subsequent addition of retinol to r*Rs*-FAR-1+cis-parinaric acid and the addition of an oleic acid solution to r*Rs*-FAR-1+cis-parinaric acid+retinol were examined.

SDS-PAGE showed that the recombinant *Rs*-FAR-1 was purified effectively, resulting in only one approximately 20 kDa band (Fig. 2), which was consistent with the theoretical molecular mass (20,814.1 Da) of *Rs*-FAR-1. The purified *Rs*-FAR-1 was found to bind the fatty acids DAUDA, cis-parinaric acid and retinoic acid (Fig. 3). The fluorescence intensity was low when r*Rs-*FAR-1, DAUDA or retinoic acid was in the buffer alone but increased when r*Rs-*FAR-1 bound to DAUDA or retinoic acid, shifting the peak emission. These results indicate that the retinoid and fatty acid binding sites are congruent, overlapping, or interactive, and the addition of oleic acid can competitively displace cis-parinaric acid.

Fluorescence titration experiments were performed to determine the binding affinity of DAUDA, retinoic acid and cis-parinaric acid. Fig. 3 shows the typical saturation binding curves for each of the three fluorescent ligands. The results indicated that *Rs*-FAR-1 is a functional protein that is able to bind fatty acids and retinoids *in vitro*.

### Expression of *Rs-far-1* and localization of *Rs-*FAR-1

**Fig 4:**
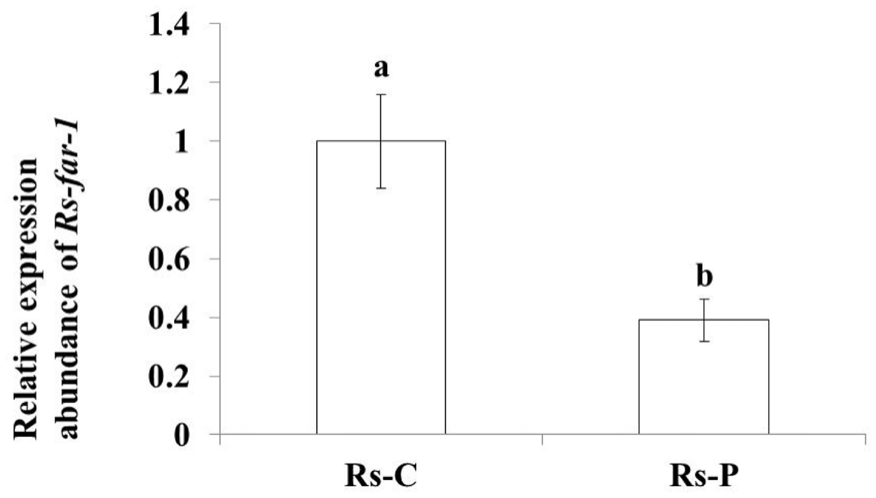
Expression levels of *Rs-far-1* in the *Radopholus similis Rs-*C and *Rs-*P populations. Bars indicate the standard errors of mean data (n = 3), and different letters indicate significant differences (*p*<0.05) between treatments. *Rs-*P, *Rs-*C: *Rs-*P and *Rs-*C populations of *R*. *similis*, collected from the roots of the ornamental plants *Philodendron cv* Green Emerald and *Calathea makoyana*, respectively.

**Fig 5:**
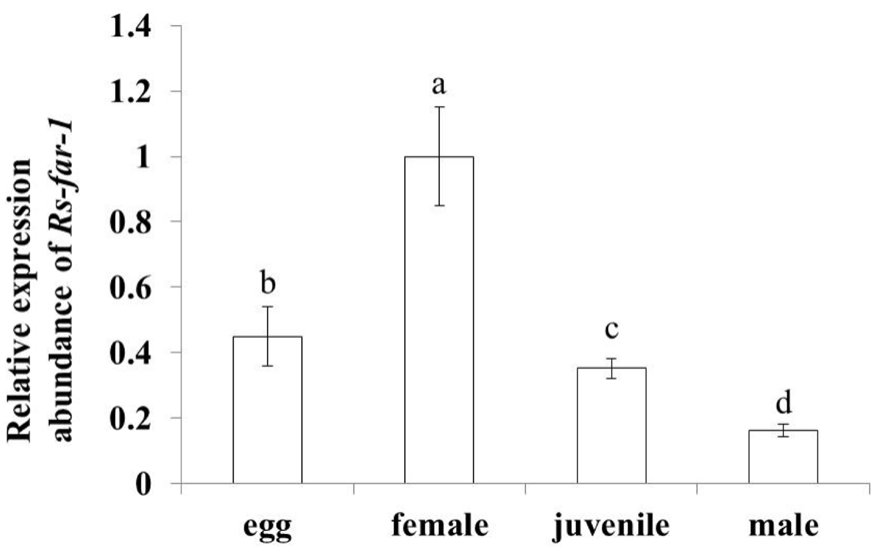
Expression levels of *Rs-far-1* in eggs, females, juveniles and males of *Radopholus similis*. Bars indicate the standard errors of mean data (n = 3), and different letters indicate significant differences (*p*<0.05) between treatments.

The qPCR results showed that the expression of *Rs-far-1* in *Rs*-C was approximately 2.5 times greater than the expression observed in *Rs*-P (Fig. 4). The *Rs-far-1* mRNA transcript was present in all developmental stages. The highest transcript levels were observed in females, while expression in the eggs, juveniles and males corresponded to 45%, 35.2%, and 16.3% of the expression observed in females, respectively (Fig. 5).

**Fig 6:**
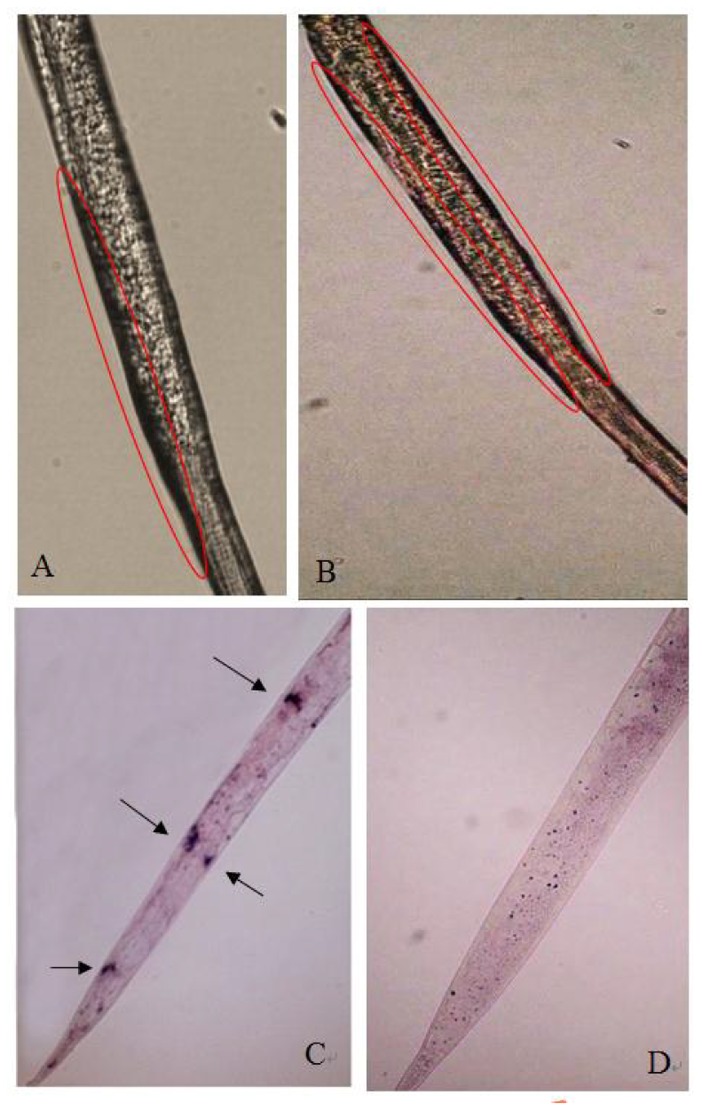
*In situ* localization of *Rs*-FAR-1 in *Radopholus similis*. A, B, C:*In situ* hybridization with the antisense ssRNA probe-signals were observed in cuticle of *R*. *similis*; D: *In situ* hybridization with the sense ssRNA probe. Red circles and arrows indicate the *In situ* hybridization signals; panels A, B and D show females and panel C shows larvae.

The results of *In situ* hybridization showed that *Rs-far-1* mRNA was present in the hypodermis of *R*. *similis* (Fig. 6A, B, C), and no hybridization signal was detected in the nematodes after incubation with the control sense probe (Fig. 6D).

### *Rs-far-1* silencing efficiency and influence on the reproduction and pathogenicity of *R*. *similis*

**Fig 7:**
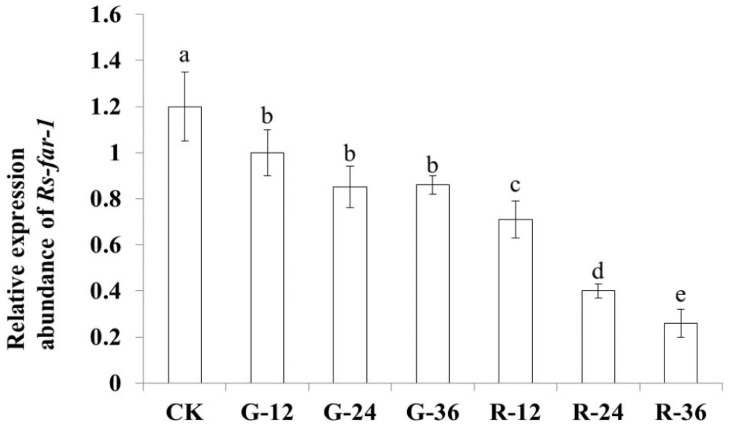
Expression of *Rs-far-1* in *Radopholus similis* under different treatments. CK: blank control;G-12, G-24 and G-36: *R*. *similis* were soaked in *gfp* dsRNA for 12 h、24 h and 3 h;R-12, R-24 and R-36: *R*. *similis* were soaked in dsRNA of *Rs-far-1* for 12 h, 24 h and 36 h. Bars indicate the standard errors of mean data (n = 3), and different letters indicate significant differences (*p*<0.05) between treatments.

qPCR was used to detect *Rs-far-1* expression after treatment with *Rs-far-1* dsRNA for 12 h, 24 h and 36 h, and it was found that compared with the *gfp* dsRNA treatment, *Rs-far-1* expression was decreased significantly, by 29.2%, 43.5% and 61.3%, respectively (*p*<0.05) (Fig. 7). The *Rs-far-1* mRNA expression level decreased under increasing incubation times with *Rs-far-1* dsRNA. Compared with *Rs-far-1* expression in untreated nematodes, *Rs-far-1* expression in nematodes that were treated with *gfp* dsRNA for 12 h, 24 h and 36 h was significantly decreased (*p*<0.05), but there was no significant difference between different time points under treatment with *gfp* dsRNA.

**Fig 8:**
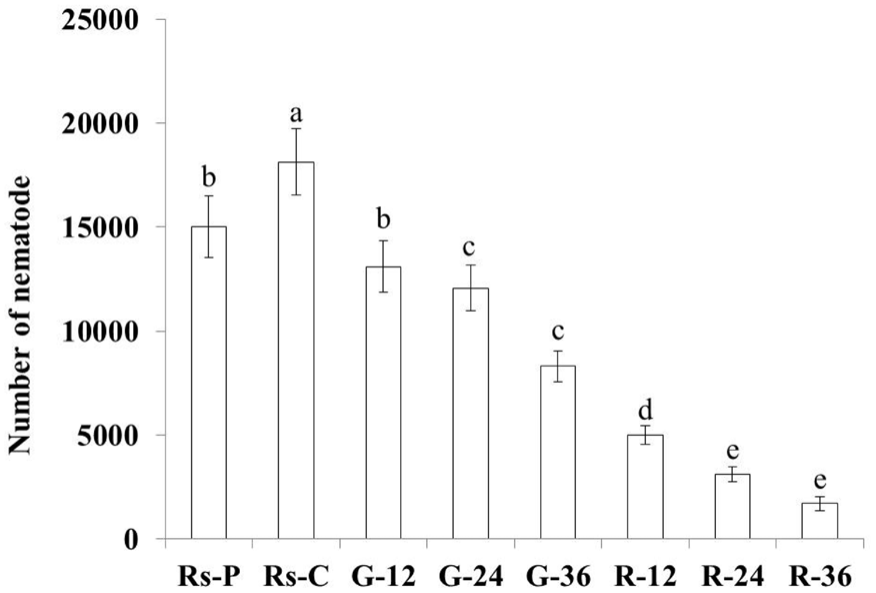
Number of nematodes separated from carrot disks inoculated with *Radopholus similis* subjected to different treatments for 56 d. *Rs-*P, *Rs-*C:inoculated with the *Rs-*P and *Rs-*C populations of *R*. *similis* collected from the roots of the ornamental plants *Philodendron cv* Green Emerald and *Calathea makoyana*, respectively;R-12, R-24 and R-36: number of nematodes isolated from carrot disks inoculated for 56 d with *R*. *similis* soaked in *Rs-far-1* dsRNA for 12 h, 24 h and 36 h; G-12, G-24, G-36: *R*. *similis* were soaked in *gfp* dsRNA for 12 h, 24 h and 36 h. Bars indicate the standard errors of mean data (n = 5), and different letters indicate significant differences (*p* <0.05) between treatments.

The effect of gene silencing on nematode reproduction was studied through inoculation with treated and untreated nematodes grown on a carrot callus for 56 days. *Rs*-C nematodes treated with *Rs-far-1* dsRNA for 12 h, 24 h, and 36 h presented significantly lower reproduction than the untreated *Rs*-C and *Rs*-P nematodes and the *Rs*-C nematodes treated with *gfp* dsRNA (Fig. 8). Furthermore, the reproduction of nematodes treated with *Rs-far-1* dsRNA decreased as the exposure time increased; there was a significant difference (*p*<0.05) between the 12 h and 24 h treatment times, whereas the 24 h and 36 h treatment times showed no significant difference (*p*>0.05) (Fig. 8). Therefore, the reproduction of nematodes treated for 12 h with *Rs-far-1* dsRNA was decreased significantly (*p*<0.05), and the reproduction of the untreated *Rs*-C nematodes was significantly greater than that of the untreated *Rs*-P nematodes (*p*<0.05) (Fig. 8).

**Fig 9:**
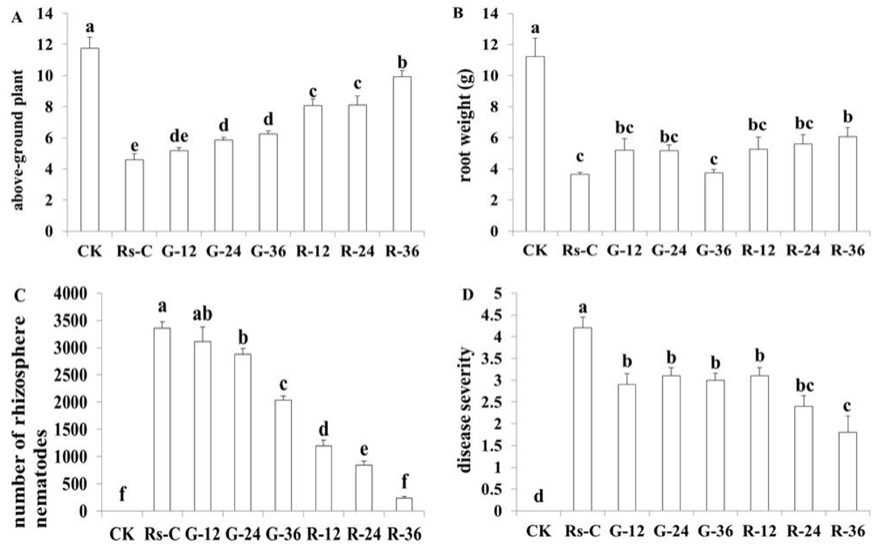
Physiological status (above-ground plant weight, root weight and disease severity) and nematodes in the rhizosphere of *Anthurium andraeanum* infected with *Radopholus similis* subjected to different treatments after 56 d. Bars indicate the standard errors of mean data (n = 5), and different letters indicate significant differences (*p*<0.05) between treatments. A: above-ground plant weight (g); B: root weight (g); C: number of rhizosphere nematodes; D: disease severity. CK: blank control; *Rs-*P, *Rs-*C: infected by the *Rs-*P and *Rs-*C populations; R-12, R-24, R-36 and G-12, G-24, G-36: *Anthurium andraeanum* infected with *R*. *similis* treated with of *Rs-far-1* or *gfp* dsRNA for 12 h, 24 h and 36 h.

Anthurium plants were inoculated with *Rs*-C nematodes that were treated with *Rs-far-1* dsRNA or *gfp* dsRNA, or with untreated *Rs*-C and *Rs*-P nematodes. After the 56-day infection period, the above-ground parts of the plants and the roots were weighed and compared. The results (Fig. 9A and B) showed that the anthurium plants inoculated with *Rs*-C nematodes treated with *Rs-far-1* dsRNA for 36 h exhibited the greatest above-ground and root weights, and both the above-ground weights and root weights were significantly different (*p*<0.05) from those recorded in the untreated *Rs*-C inoculation treatment and the *gfp* dsRNA treatment. Anthurium growth showed no significant difference in the *Rs-far-1* dsRNA-treated plants that were treated for 12 h and 24 h (*p*>0.05), and the root weights recorded in the two treatments did not show any significant differences (*p*<0.05) compared with the other treatments, although the above-ground weights obtained in the two RNAi treatments were significantly different (*p*<0.05) from those in the untreated *Rs*-C and *gfp* dsRNA inoculation treatments. Comparison of the above-ground and root weights observed in the other treatments revealed that only the nematodes inoculated with *gfp* dsRNA for 24 h and 36 h showed a significant difference compared with the untreated *Rs*-C inoculation.

The number of nematodes in the rhizosphere inoculated with the *Rs*-C nematodes treated with *Rs-far-1* dsRNA for different times was significantly lower (*p*<0.05) than in the anthurium plants inoculated with untreated *Rs*-C or *gfp* dsRNA-treated nematodes for the same duration. Additionally, disease severity in the anthurium plants inoculated with the *Rs-far-1* dsRNA-treated nematodes for 12 h, 24 h and 36 h was significantly lower (*p*<0.05) than in the untreated *Rs*-C inoculation. Among the RNAi treatments, the 36 h treatment with *Rs-far-1* dsRNA resulted in the lowest disease severity among the inoculation treatments, with the exception of the 24 h *Rs-far-1* dsRNA treatment. There were no significant differences (*p*>0.05) among the other inoculation treatments. These results indicate that the pathogenicity of the *Rs*-C nematodes treated with *Rs-far-1* dsRNA was decreased, especially after 36 h of treatment (*p*<0.05).

### Effect of *Rs-far-1* expression on AOS expression in *A*. *thaliana*

qRT-PCR was performed to measure AOS expression in *A*. *thaliana* inoculated with *Rs*-C nematodes for 12 h, 24 h, 36 h, 48 h, 60 h and 72 h. Compared with untreated *A*. *thaliana* (CK), AOS expression was increased significantly, by 50% (*p*<0.05), at the beginning of the infection process (at 12 h) and by 15% at 24 h, which was not a significant difference (*p*>0.05). However, AOS expression decreased significantly (*p*<0.05), to below the expression level in CK at 36 h, and there was no significant difference between the inoculation treatments (*p*>0.05).

**Fig 10:**
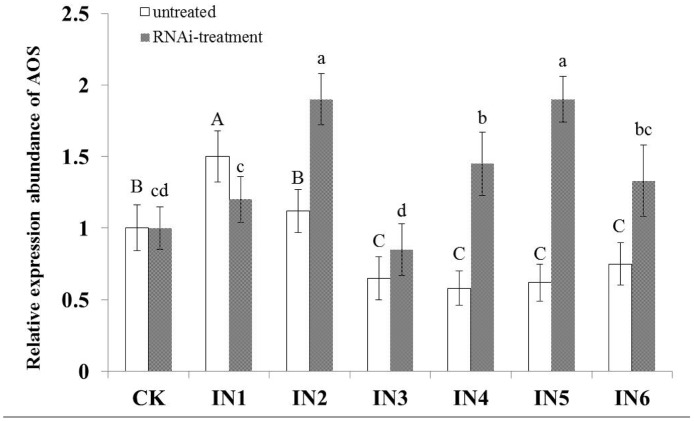
Expression of the AOS gene in *Arabidopsis thaliana* infected with *Radopholus similis* after different times. Bars indicate the standard errors of mean data (n = 3); different capital letters indicate significant differences (*p*<0.05) between untreated *R*. *similis*, and different lowercase letters indicate significant differences (*p*<0.05) between different *R*. *similis* RNAi treatments. CK: blank control; IN-1 to IN-6: infected by untreated and RNAi-treated *R*. *similis* after 12 h, 24 h, 36 h, 48 h, 60 h and 72 h. AOS: allene oxide synthase.

AOS expression in *A*. *thaliana* inoculated with *Rs-far-1* RNAi-treated *Rs*-C nematodes for 12–72 h was evaluated every 12 h using qRT-PCR. The results (Fig. 10) indicated that compared with untreated *A*. *thaliana* (CK), AOS expression did not increase significantly (*p*>0.05) at the beginning of the infection process (12 h), but at 24 h, AOS expression had increased significantly, by 100% (*p*<0.05). However, AOS expression then decreased and was not significantly different from that in CK at 36 h. Subsequently, AOS expression increased and was significantly higher than that in CK at 48 h and 60 h (there was also a significant difference between 48 h and 60 h), and at 72 h, AOS expression returned to the same level as in CK.

Based on these results, AOS expression in *A*. *thaliana* infected with untreated and RNAi-treated nematodes increased at the beginning of the infection process and then decreased back to levels that were similar to those in CK. Subsequently, AOS expression in *A*. *thaliana* infected with untreated nematodes decreased to levels that were significantly lower than those in CK. However, AOS expression in *A*. *thaliana* infected with RNAi-treated nematodes increased to levels that were significantly higher than those in CK. This effect may have been influenced by the efficiency of RNAi, which may have decreased over time. All of these results suggest that *Rs-far-1* may regulate AOS expression in *A*. *thaliana*.

## Discussion

This study is the first to clone the FAR gene *Rs*-*far-1* and to report that the expressed protein is located in the hypodermis of *R*. *similis*. We demonstrated that *Rs*-FAR-1 binds to fatty acids and retinoids *in vitro*, which may play a role in the development and pathogenicity of *R*. *similis* and enable *Rs*-FAR-1 to be involved the plant defense system.

In this study, we observed that the expression of *Rs-far-1* in *Rs*-C, which shows higher reproduction and pathogenicity, was approximately 2.5 times greater than in *Rs*-P, which shows lower reproduction and pathogenicity, suggesting that *Rs-far-1* may be associated with the reproduction and pathogenicity of nematodes. FARs are capable of absorbing and transferring fatty acids and retinoids from host plants [33,34], and these bound lipids are used for nematode metabolism, which is beneficial to the nematode but injures the host plant, as cell differentiation and tissue repair are affected by decreasing fatty acid and retinoid concentrations. Additionally, because FARs bind to linolenic and linoleic acid from the host plant, the synthesis of jasmonic acid (JA), which is the signal transducer in the host defense response, is hindered, reducing the plant defense response and increasing disease severity [24,35]. Therefore, nematodes with higher *far-1* expression levels acquire more fatty acids and retinoids for their metabolism and further decrease the JA concentration, ultimately leading to a decreased defense response in the host plant and higher pathogenicity of the nematodes. This mechanism is further supported by evidence regarding the relationship between *Rs-far-1* and the pathogenicity of *R*. *similis* subjected to *Rs-far-1* RNAi.

In this study, *Rs-far-1* mRNA expression was found to be highest in females and lowest in males, and it was higher in eggs than in juveniles, which is consistent with findings for *Ab-far-1* from *A*. *besseyi* [26]. The expression of *Mj-far-1* from *M*. *javanica* is highest in J2, when it is significantly higher than in mature females, and its expression in J2, J3/4 and pre-J2 decreases progressively [24]. These results suggest an association between the characteristics of different FARs and the biological characteristics of each developmental stage in different types of nematodes. Nematodes not only utilize linolenic and linoleic acid bound to FARs to decrease the defense response of the host plant and achieve infection but also synthesize collagen to promote embryonic development and reproduction through binding retinoids. The expression of *far-1* was shown to be highest in the females of the migratory parasites *R*. *similis* and *A*. *besseyi* because the females are responsible for infection and reproduction. The adult females of the sedentary parasite *M*. *javanica* play key roles in reproduction, but not in infection, and *M*. *javanica* completes its development and reproduction after parasitizing the host plant; therefore, the *far-1* expression is highest in the *M*. *javanica* J2 form, which is the infective form. The expression of *far-1* was observed to be significantly higher in the eggs than that in the juveniles of both *R*. *similis* and *A*. *besseyi*, and *Mj-far-1* expression is significantly lower in the eggs than in the juveniles and females, possibly because the fecundity of *R*. *similis* and *A*. *besseyi* is lower than that of *M*. *javanica*, and their hatchability is more crucial than that of *M*. *javanica*. These results indicate that *far-1* may participate in nematode reproduction, as confirmed by the ability of *Rs-far-1* RNAi to decrease the reproduction of *R*. *similis*, which was also observed for *Ab-far-1* from *A*. *besseyi* by Cheng et al. (2013) [26]. Although the expression of *Rs-far-1* was reduced after both *gfp* dsRNA and *Rs-far-1* dsRNA treatment, the influence of *gfp* dsRNA was significantly weaker than that of *Rs-far-1* dsRNA treatment. Additionally, the effect of *gfp* dsRNA was not increased through extending the soaking time, and the differences between G-12, G-24 and G-36 were not significant. The *Rs-far-1* dsRNA treatment caused the nematodes to exhibit lower hatchability and pathogenicity compared with the *gfp* dsRNA treatment, showing that the effect of *gfp* dsRNA was limited and did not increase after soaking for 24 h; similar results have been obtained in several previous studies [9,24,26].

Prior et al. [36] reported that *Gp*-FAR-1 is located in the hypodermis of *G*. *pallid* and decreases the substrate concentration in JA synthesis by binding linolenic and linoleic acid. Cheng et al. [26] reported that *Ab*-FAR-1 is located in the hypodermis and genital gland of *A*. *besseyi*. Iberkleid et al. [24] reported that *Mj*-FAR-1 is located in the cuticle layer of *M*. *javanica* and around the cuticle adjacent to the nematode after invasion. The present study revealed that *Rs-far-1* mRNA is present in the hypodermis of *R*. *similis*. The expression of *far* in the hypodermis may help nematodes to quickly utilize fatty acids and retinoids from the host and the environment to maintain autologous metabolism. Additionally, *far* expression in the hypodermis may help nematodes to neutralize the plant defense system and to complete the infection process by utilizing linolenic and linoleic acid and decreasing JA synthesis in the host plant.

According to the evolutionary relationships between different FAR-1s, Tylenchida plant-parasitic nematodes can be classified into *Meloidogyne*, *Radopholus*, *Pratylenchus*, *Globodera*, and *Heterodera*, and these 5 genera can be further classified into migratory endoparasitic nematodes and sedentary parasitic nematodes based on their parasitic behavior. Cheng et al. [26] divided *Ab*-FAR-1 and 25 other FAR proteins from 16 nematode species into 6 groups: Spirurida, Ascaridida, Tylenchida, Aphelenchida, Strongylida, and Rhabditida. Iberkleid et al. [24] classified the FAR proteins from different living nematodes into five main clusters (A to E), which fell into the following categories: FAR proteins from the free-living nematode *C*. *elegans* (clade A), animal-parasitic nematodes (clade B), root-knot nematodes (clade C), endomigratory plant-parasitic nematodes (clade D) and cyst nematodes (clade E). In the present work, we speculated that *Rs-far-1* regulates AOS expression. To the best of our knowledge, this is the first study to reveal the association between *Rs-*FAR-1 and the JA pathway of *A*. *thaliana*. Iberkleid et al. [24] reported that overexpression of *Mj-far-1* in the hairy roots of tomatoes caused significant downregulation of the proteinase inhibitor *Pin2* (accession no. L21194) and γ-thionin (accession no. AJ133601.1) and inhibited the defense response. We propose that *Rs-far-1* may be a crucial gene that participates in resisting the defense response of host plants.
